# High vaccination coverage is associated with low epidemic level of seasonal influenza in elementary schools: an observational study in Matsumoto City, Japan

**DOI:** 10.1186/s12879-018-3025-9

**Published:** 2018-03-13

**Authors:** Mitsuo Uchida, Minoru Kaneko, Yoshihiko Hidaka, Hiroshi Yamamoto, Takayuki Honda, Shouhei Takeuchi, Masaya Saito, Shigeyuki Kawa

**Affiliations:** 1 Center for Health, Safety and Environment Management, Shinshu University, 3-1-1, Asahi, Matsumoto, Nagano, 390-8621 Japan; 2 Department of Pediatrics, Shinshu University School of Medicine, 3-1-1, Asahi, Matsumoto, Nagano, 390-8621 Japan; 3 The First Department of Internal Medicine, Shinshu University School of Medicine, 3-1-1, Asahi, Matsumoto, Nagano, 390-8621 Japan; 4 Department of Laboratory Medicine, Shinshu University School of Medicine, 3-1-1, Asahi, Matsumoto, Nagano, 390-8621 Japan; 5 Department of Public Health, Faculty of Medicine, University of Miyazaki, 1-1, Gakuenkihanadainishi, Miyazaki, Miyazaki 889-2192 Japan; 6 Institute of Statistical Mathematics, 10-3, Midorimachi, Tachikawa, Tokyo, 190-8562 Japan

**Keywords:** Schoolchildren, Epidemiology, Seasonal influenza, Reproduction number, Infection control measure

## Abstract

**Background:**

Influenza virus transmission may be prevented by infection control measures, including vaccination, wearing a mask, gargling with water, and hand washing. It is unclear, however, whether these measures affect influenza epidemics in school settings.

**Methods:**

A prospective epidemiological survey in all public elementary schools in Matsumoto City, Japan, during the 2014/2015 season evaluated the number of diagnosed patients in each school and calculated the reproduction number of schoolchildren. At the end of the prospective survey, a cross-sectional survey evaluated the implementation of infection control measures in these schools. Both results were combined and associations among infection control measures including vaccination, mask wearing, hand washing, water gargling, and epidemic level were evaluated.

**Results:**

Of the 13,217 schoolchildren in 29 schools, 2548 were diagnosed with seasonal influenza. A significant negative association was observed between vaccination coverage and reproduction number at each school, but not between other infection control measures and the reproduction number. A regression curve with exponential function was most predictive. At 0% vaccination, the reproduction number was estimated to be 1.39.

**Conclusion:**

These findings provide evidence that high vaccination coverage was associated with reduced epidemic levels in schools and suggest the need for increased vaccination of schoolchildren.

## Background

Seasonal influenza is a common infectious disease that usually spreads among children. Although several influenza control measures have been implemented in children to reduce or prevent virus transmission [1], seasonal influenza shows yearly outbreaks and remains a major disease throughout the world [2]. These yearly outbreaks are thought to be caused not only by continuous evolution of the influenza virus [3], but by the behavior and activities of children. Specific contact patterns in children can affect virus transmission [4].

Two categories of influenza control measures are generally used to prevent influenza virus transmission: pharmaceutical and non-pharmaceutical interventions [5]. The primary form of pharmaceutical intervention is vaccination, which has been shown to prevent severe outcomes caused by influenza viruses [2]. Vaccination has a direct effect in individuals, strengthening their level of immunization, as well as having an indirect effect in patients, including the induction of herd immunity [6, 7].

Non-pharmaceutical interventions (NPIs) are thought to be useful when vaccination is insufficient or inappropriate. NPIs to prevent virus transmission can include wearing a mask [8, 9], hand washing [10, 11] and gargling with water [12]. While protective effects of NPIs are expected, studies assessing the efficacy and effectiveness of these methods have shown inconsistent results [13, 14], primarily due to differences in sample sizes and study settings.

Although many experimental and epidemiological studies have evaluated the association between interventional measures and influenza prevention, fewer studies have assessed the efficacy and effectiveness of these measures in school settings. Collective implementation of these influenza control measures varies among schools, with these variations possibly affecting the epidemic level of influenza in these schools. Differences in the epidemic level of seasonal influenza among schools may be clarified by epidemiological studies of all schools within a specific community, allowing factors affecting these differences to be determined.

An observational epidemiological study was therefore performed, involving children enrolled at all schools in Matsumoto City, Japan, during the 2014/2015 seasonal influenza period [15, 16]. Information was obtained about the diagnosis of seasonal influenza and individual infection control measures among children in all elementary schools. Although these previous studies showed associations between vaccination and mask wearing and prevention of influenza symptom onset at an individual level, the question of whether those measures also prevent epidemic levels in each school remains. In this study, the associations between collective infection control measures and the epidemic level at these schools were evaluated.

## Methods

### Study subjects

This observational epidemiological study assessed seasonal influenza during the 2014/2015 season among elementary schoolchildren in Matsumoto City, Japan [15, 16]. This suburban city is located in the center of Japan and has a population of about 240,000 individuals. The city contains 29 public elementary schools with 13,217 schoolchildren aged 7–12 years, corresponding to 97% of the school-aged population. The study subjects include all these children, not their parents/guardians or teachers.

### Two step survey

Detailed study methods were described previously [15, 16]. This study utilized a two-step survey. In brief, the first survey, performed from October 2014 to the end of February 2015 [15], was designed to evaluate influenza epidemic dynamics in schoolchildren throughout an entire community. In Japan, the parents or guardians of schoolchildren who are symptomatic and have been diagnosed with seasonal influenza at medical institutions must submit a certified note to the child’s school. This note is initially given by the school to the parent or guardian when they inform the school that their child has been diagnosed as influenza. These children are not allowed to return to school until recovery to prevent transmission of the virus. The certified note includes information about the diagnosis at medical institutions. In this study, schoolchildren diagnosed at medical institutions were defined as having influenza.

At the time the note was distributed to the child’s household, a questionnaire was also distributed. The questionnaire included questions about date of fever onset and duration of fever (description); diagnosis at medical institution (yes/no) and, if yes, influenza subtype (type A, type B, unknown), and number of days not attending school (description). Responses were obtained from 2548 children, corresponding to 96.1% of all children who received a note from school about influenza diagnosis. Of the 29 schools, one was confirmed as having no cases of influenza. According to this first survey, it gave information of epidemic levels in respective schools.

The second survey was performed at the end of February 2015 and involved all schoolchildren in Matsumoto City [16]. This second survey was designed to assess the implementation of infection control measures reported by children with and without influenza to clarify the factors associated with onset of influenza at the individual and community level. Questionnaire in the second survey included questions on experience with the 2014/2015 seasonal influenza (yes/no), and, if yes, the data of onset (calendar month) and diagnosis by a medical institution (yes/no). A question on vaccination was included (yes/no), and, if yes, the calendar month. Subjects were regarded as vaccinated if they received at least one dose of vaccine. Questions were also asked about the use of NPIs anywhere or anytime, including habitual mask wearing (yes/no), habitual water gargling (yes/no) and habitual hand washing with water (yes/no). Frequencies of NPIs were not evaluated and only experiential information was obtained. Questionnaires were returned from 11,390 (86.2%) of the 13,217 children. After excluding questionnaires with missing data, data from 10,524 children were analyzed. The second survey determined the proportions of each infection control measure applied.

The second-hand data of the above two reports were combined and divided into school units in this study. In each school, the association between a proportion of individual infection control measures and respective epidemic level was evaluated.

### Calculation of epidemic levels at individual schools

Influenza is a communicable disease which differs from other diseases that occur independently. An infection scale which measures or predicts transmission of the disease among a population is required to consider the epidemic level in each school. The infectiousness of a disease can be determined by calculating its basic reproduction number (*R*_*0*_), allowing an epidemic level to be estimated. The *R*_*0*_ of seasonal influenza is generally around 1.3 [17]. Reproduction number can be estimated using a mathematical SIR model and the number of infected individuals [18] using the formula:

$$ {R}_0=-\ln \left(1-p\right)/p $$

where *p* is the proportion of final size of infected individuals.

Because this study was based on questionnaires, subjects who were asymptomatic or had mild symptoms and who were not diagnosed at medical institutions were not evaluated. Moreover, because infection control measures, such as vaccination or NPIs, were implemented at the level of individuals, a partially effective reproduction number, which considers the effect of several infection control measures, was calculated. A simple reproduction number (*R*), based on the number of diagnosed children was applied.

$$ R=-\ln \left(1-p\right)/p $$

where *p* is the proportion of children diagnosed at each school.

### Herd immunity

Determination of a basic reproduction number allows calculations of minimal vaccination coverage to prevent the outbreak of an epidemic. This coverage is generally associated with herd immunity [6, 19]. A least coverage “*P*” of vaccination is usually calculated as:

$$ P=1\hbox{--} \left(1/{R}_0\right) $$

Furthermore, if vaccine efficacy was imperfect, the formula was divided as efficacious proportion ε.

$$ P=\left\{1\hbox{--} \left(1/{R}_0\right)\right\}/\varepsilon $$

In this study, the required herd immunity level was calculated based on *R*_*0*_ and ε.

### Statistical analysis

If the *R*_*0*_ is stable, greater implementation of infection control measures may decrease the epidemic level at a school and lower the *R*. Although our previous study showed that some NPIs had slight correlations with each other at the individual level [16], in the present study, we aimed to determine whether there was any association between the proportion of these measures adopted in each school and the epidemic level. Therefore, the association between infection control measures and *R* was assessed by correlation independently. Because data were not distributed normally, Spearman’s correlation analysis was used, with *p* < 0.05 defined as statistically significant, with significant correlations further analyzed by regression analysis. Three types of regression curves were drawn. Linear models were calculated with the least squares method. In addition, a maximum log-likelihood method was used to develop a generalized linear model (GLM) with exponential function. In the latter method, the *R* for gamma distribution was fitted for estimation. Akaike’s Information Criterion (AIC) values were calculated for prediction and regression curves, with the most predictive model defined as that with the lowest AIC. Statistical analyses were performed using SPSS ver22.0 (CA, USA) and R software (ver. 3.3.0).

## Results

**Fig. 1:**
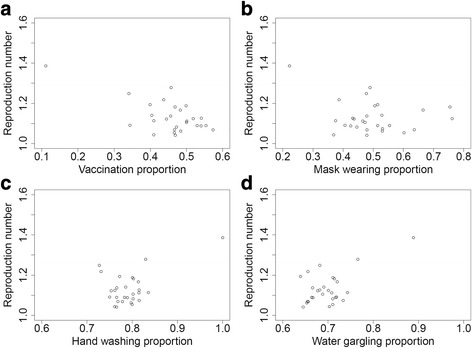
Association between infection control measures and reproduction number. **a** The proportion of subjects vaccinated showed a significant negative correlation with reproduction number (*ρ* = −0.413, *p* = 0.029). However, **b** mask wearing, **c** hand washing and **d** gargling with water were not significantly associated with reproduction number

The descriptive epidemiology of the epidemic has been reported previously [15]. In brief, the seasonal influenza epidemic started at the end of November 2014 and finished at the end of February 2015. The first peak occurred at the end of December 2014, and after winter recess, second peak occurred in the fifth week of 2015. In first survey, responses were received for 2548 children. Almost all of these children were diagnosed at medical institutions using rapid diagnostic kits, with a few diagnosed by having symptoms of an influenza like illness. None was diagnosed by laboratory confirmation. Of the infected patients, 95.0% were diagnosed with type A influenza, in agreement with a national report that influenza AH3 had spread throughout this influenza season in Japan [20]. The vaccine used in Japan during this season was directed against three strains: A/California/7/2009(H1N1)pdm09, A/New York/39/2012(H3N2) and B/Massachusetts/2/2012 [20]. Although some classes were closed due to the epidemic, entire schools were not closed. *R*s and the proportions of infection control measures at each school were calculated (Table 1). As one school had no affected students, *R*s were calculated for the other 28 schools [15]. These *R*s ranged from 1.04 to 1.39. Consequently, the proportions of infection control measures were calculated in each school according to the second survey. When correlations between *R*s and each infection control measure were calculated (Fig. 1a-d), a significant negative correlation was observed between vaccination coverage and *R* (ρ (rho) = − 0.413, *p* = 0.029). In contrast, *R*s did not correlate significantly with the other infection control measures, including wearing masks (*ρ* = − 0.056, *p* = 0.776), hand washing (*ρ* = 0.105, *p* = 0.594) and gargling (*ρ* = 0.257, *p* = 0.187).

**Table 1:** Relationship between reproduction number (*R*) and infection control measures in 29 schools in Matsumoto City, Japan

School Number	Number of Children	Number Infected	*R*	Vaccination	Wearing mask	Hand washing	Gargling
1	382	30	1.04	0.47	0.48	0.77	0.64
2	931	107	1.06	0.48	0.53	0.80	0.65
3	37	6	1.09	0.34	0.41	0.75	0.72
4	167	56	1.22	0.44	0.39	0.73	0.66
5	14	7	1.39	0.11	0.22	1.00	0.89
6	410	86	1.12	0.44	0.44	0.75	0.68
7	662	98	1.08	0.47	0.45	0.80	0.71
8	791	187	1.14	0.40	0.53	0.79	0.69
9	890	173	1.11	0.50	0.47	0.81	0.71
10	305	48	1.09	0.54	0.43	0.79	0.67
11	904	91	1.05	0.47	0.60	0.80	0.71
12	154	57	1.25	0.34	0.48	0.73	0.68
13	453	61	1.07	0.62	0.53	0.82	0.73
14	663	106	1.09	0.55	0.55	0.77	0.66
15	338	54	1.09	0.53	0.51	0.78	0.72
16	602	139	1.14	0.46	0.48	0.77	0.67
17	650	120	1.11	0.50	0.47	0.80	0.69
18	259	55	1.12	0.52	0.76	0.76	0.70
19	423	53	1.07	0.57	0.48	0.78	0.66
20	127	51	1.28	0.46	0.49	0.83	0.77
21	30	0	N/A	0.52	0.41	0.70	0.52
22	352	29	1.04	0.41	0.37	0.76	0.70
23	889	259	1.18	0.47	0.75	0.80	0.71
24	335	91	1.17	0.48	0.67	0.81	0.72
25	364	46	1.07	0.47	0.64	0.77	0.65
26	438	134	1.19	0.40	0.51	0.77	0.64
27	508	100	1.11	0.41	0.38	0.84	0.74
28	703	210	1.19	0.50	0.51	0.80	0.71
29	436	94	1.13	0.54	0.43	0.82	0.68

**Fig. 2:**
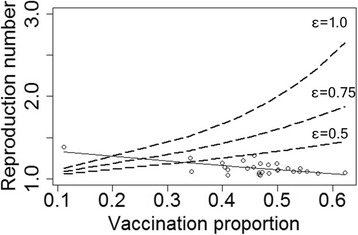
Generalized linear regression model between vaccination coverage and reproduction number in schools. A generalized linear regression model (solid line) was more predictive of reproduction number (AIC = −74.55) than other regression curves. High vaccine coverage was associated with lower reproduction numbers among schools. Required vaccine coverage was determined in three vaccine efficacy levels (ε = 1.0, 0.75 and 0.5). As efficacy became smaller, the cross point between the regression curve and the dashed line moved rightwards, indicating that lower vaccine efficacy requires higher vaccine coverage in elementary schools

Regression model was used to show the relationship between vaccination coverage and *R*s (Fig. 2). The regression curve with the solid line was obtained from observed data, showing a negative association between high vaccine coverage and *R*s. Using the GLM method, a regression curve with an exponential function (y = exp(0.3314–0.452×)) was most predictive, with the lowest AIC (− 74.55). Other predictive curves using a quadratic equation y = 0.9764 × ^2^–1.2861× + 1.5091 (AIC -73.48) or a linear equation y = − 0.5445× + 1.383 (AIC -73.14) were inferior. The regression curve showed that, if none of the schoolchildren at a school was vaccinated (vaccination rate 0%), the *R* at that school would be 1.39. Additionally, required vaccine coverage lines were plotted in the figure. The vaccine efficacy “ε” was hypothesized with 1.0, 0.75 and 0.5. When the efficacy value became lower, the dashed line was pushed down and a cross point between the regression curve and the required vaccine coverage curve moved rightwards. If the efficacy was 0.5, the required vaccine coverage was indicated as around 35%.

## Discussion

Although seasonal influenza infection was common among schoolchildren, the reasons underlying differences in the influenza epidemic level among schools have been unclear. We previously showed that the number of schoolchildren at each school did not correlate significantly with epidemic level [15]. As a follow-up, we assessed whether implementation of infection control measures correlated with influenza epidemic level at each school. Implementation of infection control measures by children was included in questionnaires, allowing the proportion of children implementing these measures to be calculated for each school. Of the four infection control measures analyzed, only vaccination showed a significant negative correlation with reproduction number, whereas the other measures, including mask wearing, hand washing, and gargling with water, were not.

Many studies have found that seasonal influenza vaccines is efficacious in children [21]. Although seasonal influenza was researched in school settings, the association between vaccination and influenza epidemic level in all schools of a specific community had not been determined. This lack of information may hamper the implementation of a vaccination policy at the school level. Our findings, showing a negative association between vaccine coverage and influenza epidemic level at schools, may help to promote a vaccination plan.

Two hypotheses may explain the negative association between vaccine coverage and influenza epidemic level. First, vaccine coverage may simply reduce the size and spread of an epidemic. Second, higher vaccine coverage may tend to induce herd immunity [6, 19] in school organization. Herd immunity should always be considered when evaluating communicable diseases epidemiologically. Especially, a large sample size study is more likely to show any strong effects of herd immunity [22]. Although this study, being cross-sectional in design, could not determine the contribution of herd immunity, this large sample size study found that high vaccination coverage was associated with low reproduction number.

In general, the accuracy of reproduction number *R*_*0*_ is only precise if it is calculated for a large homogeneous population, and since the known population of school-age children in our study is also surrounded by an unknown population of peers, neighbors and family members among whom influenza transmission is possible, our reproduction number may over or underestimated, compared with such a number in a controlled experiment. However, especially in epidemiological [15] and mathematical modeling fields [23], this use of the reproduction number at schools is accepted practice in assessing epidemic levels. Moreover, further research is required to determine the reliability and validity of using reproduction numbers in such public health studies.

Then, we estimated that the reproduction number when vaccination coverage was 0% would be 1.39, similar to the basic reproduction number (1.3) of seasonal influenza [17]. The reproduction number of 1.39 was based on symptomatic and diagnosed patients in school settings. This number may be useful in schools when only diagnosed patients are evaluated. In addition, the required vaccine coverage which was calculated by the reproduction number was determined in 3 levels of vaccine efficacy. Influenza vaccine sometimes does not fully match the actual seasonal influenza strain. In fact, the strain which had spread in Japan was not matched with the vaccine used in this season [20]. If the efficacy becomes smaller, the required vaccine coverage should be increased. As a result of this study, the required vaccine coverage which was calculated by estimated regression curve was around 35% when efficacy was 0.5. This suggests that not only vaccine coverage but vaccine efficacy are important factors to determine vaccine policy in school settings.

NPIs are generally used to prevent influenza transmission [1]. Some experimental studies have shown that NPIs, including wearing a mask, gargling and hand washing, were effective [11, 12, 24]. We investigated the effects of NPIs at the individual level in our previous observational study and found some protective effects in face mask and vaccination with influenza diagnosis among subjects using a regression model [16]. However, the association between collective NPI implementation proportion and epidemic levels in schools remains unclear. To evaluate associations between epidemic level and these measures, *R* was calculated in this study. Some studies have reported that public health emphasis [25], and social networking may affect influenza transmission [23]. In the present study, we investigated the association between several NPI proportions and epidemic level in school settings, however, we did not find any evidence. This disparity may be because NPIs might have more protective effects at the individual level with these effects difficult to show herd effect in epidemic levels in schools than vaccination. Moreover, NPIs in this study were evaluated by questionnaire, therefore, a more subjective answer could be obtained than vaccination. Future studies should attempt to quantitatively evaluate the use of NPIs.

This study had several limitations. First, utilization of the reproduction number is a limitation to the extent that its accuracy or precision is not assured as discussed above. Because this study was based on a self-administrated questionnaire, blood samples were not obtained and recall bias could not be ruled out. A high proportion of patients infected with influenza virus are asymptomatic [26]. Further, the number of schoolchildren was not uniform so the reproduction number might not be stable. Because these issues may affect its accuracy, the reproduction number should be treated with caution. Second, in Japan, children < 13 years old are required to be vaccinated twice during each influenza season [27]. However, the number of vaccinations was not evaluated in this study. Further, because NPIs were not evaluated quantitatively, but only qualitatively, the rates of implementation may be inaccurate. Third, because this study was conducted among elementary schoolchildren in one season, epidemic diversity could not be evaluated in other generations or during other seasons.

## Conclusions

Seasonal influenza was investigated by an observational study in all 29 elementary schools in Matsumoto City, Japan. High vaccine coverage was significantly and negatively correlated with low level of influenza epidemic among school units. However, we could not find any protective NPI effects among mask wearing, hand washing, and water gargling in this study. The results of this study may contribute to informing vaccine promotion in schoolchildren. Moreover, a longitudinal study is required to evaluate the protective effects of NPIs.

## Abbreviations

AIC: Akaike’s Information Criterion
GLM: Generalized linear model
NPI: Non-pharmaceutical intervention
*R*: Reproduction number
*R*_*0*_: Basic reproduction number
SIR model: Susceptible-infectious-recovered model
